# Regulation of ex-translational activities is the primary function of the multi-tRNA synthetase complex

**DOI:** 10.1093/nar/gkaa1183

**Published:** 2020-12-21

**Authors:** Haissi Cui, Mridu Kapur, Jolene K Diedrich, John R Yates, Susan L Ackerman, Paul Schimmel

**Affiliations:** Department of Molecular Medicine, The Scripps Research Institute, La Jolla, CA 92037, USA; Howard Hughes Medical Institute, Department of Cellular and Molecular Medicine, Section of Neurobiology, University of California, San Diego, La Jolla, CA 92093, USA; Department of Molecular Medicine, The Scripps Research Institute, La Jolla, CA 92037, USA; Department of Molecular Medicine, The Scripps Research Institute, La Jolla, CA 92037, USA; Howard Hughes Medical Institute, Department of Cellular and Molecular Medicine, Section of Neurobiology, University of California, San Diego, La Jolla, CA 92093, USA; Department of Molecular Medicine, The Scripps Research Institute, La Jolla, CA 92037, USA; Department of Molecular Medicine, The Scripps Research Institute, Jupiter, FL 33458, USA

## Abstract

During mRNA translation, tRNAs are charged by aminoacyl-tRNA synthetases and subsequently used by ribosomes. A multi-enzyme aminoacyl-tRNA synthetase complex (MSC) has been proposed to increase protein synthesis efficiency by passing charged tRNAs to ribosomes. An alternative function is that the MSC repurposes specific synthetases that are released from the MSC upon cues for functions independent of translation. To explore this, we generated mammalian cells in which arginyl-tRNA synthetase and/or glutaminyl-tRNA synthetase were absent from the MSC. Protein synthesis, under a variety of stress conditions, was unchanged. Most strikingly, levels of charged tRNA^Arg^ and tRNA^Gln^ remained unchanged and no ribosome pausing was observed at codons for arginine and glutamine. Thus, increasing or regulating protein synthesis efficiency is not dependent on arginyl-tRNA synthetase and glutaminyl-tRNA synthetase in the MSC. Alternatively, and consistent with previously reported ex-translational roles requiring changes in synthetase cellular localizations, our manipulations of the MSC visibly changed localization.

## INTRODUCTION

Macromolecular machines and multiprotein complexes facilitate efficient catalysis of their products by bringing enzymes into favorable spatial proximity (1,2). In eukaryotic cells, where reactions take place simultaneously in a crowded environment and a comparatively large volume, organization within cellular compartments is of particular importance (3). Protein synthesis is among the most essential cellular reactions and is conserved throughout evolution. Several macromolecular machines are involved in mRNA translation, with the ribosome being the most prominent (4). However, the coding information for the translation of nucleotide triplets to the correct amino acid is inherent to another class of proteins, aminoacyl-tRNA synthetases (aaRSs) (5,6). As their names imply, aaRSs catalyze the aminoacylation of transfer-RNAs (tRNAs) with their respective cognate amino acid (7).

All mammals have twenty cytosolic aaRSs, one for each amino acid (8), with the exception of glutamyl-prolyl-tRNA synthetase GluProRS. (In the following, cytosolic aaRS will be abbreviated by the 3-letter amino acid code followed by RS for protein names, and one letter code followed by *ARS1* for gene names (9).) In mammals, eight synthetases are clustered together with three adaptor proteins (AIMP1-3) to form the 1.5 MDa (10) multi-tRNA synthetase complex (MSC) (11,12). Versions of this complex are present in higher eukaryotic cells, plants, and archaea (13–18). Simpler unicellular organisms contain aaRS complexes with fewer components than multicellular organisms (16,17). The conservation of MSCs suggests a selective pressure for aaRSs to assemble. In mice, deletion of MSC adapter proteins, which result in the disassembly of the MSC, led to lethality shortly after birth (AIMP-2 deletion) (19) or is early embryonically lethal (AIMP-3 deletion) (20).

In previous work, the MSC was proposed to channel charged tRNAs directly to ribosomes to support efficient mRNA translation (21), handing substrates for protein synthesis from one complex (the MSC) to another (the ribosome). Proximity of the MSC to ribosomal proteins has been described in the archaeon *Thermococcus kodakarensis* (22) and in human cells (23). In line with this, expression of a non-MSC targeted arginyl-tRNA synthetase (ArgRS) variant failed to rescue growth defects if endogenous, genetically mutated ArgRS was inactivated by heat in CHO cells (21). In these cells, protein synthesis is impaired without marked differences in levels of charged tRNAs (21). This led to the hypothesis that the MSC provides charged tRNAs to ribosomes for efficient translation. In contrast, in another earlier study in mammalian cells, the adapter protein AIMP-2 was deleted, which led to the complete disassembly of the MSC and did not affect global protein synthesis (19,24).

In addition to facilitating mRNA translation, functions outside of protein synthesis have been described for MSC components (12). tRNA synthetases regulate cell signaling, metabolite sensing, and influence transcription and translation by binding to specific RNA and DNA sites (8,17). These functions are often independent of tRNA charging (8,25–27). For some MSC-bound aaRSs, these ex-translational functions require release of the synthetase from the MSC by a specific cue: for example, post-translational modifications on GluProRS and lysyl-tRNA synthetase (LysRS) stimulate release from the MSC for new functionalities (28–30). Here, the MSC acts as a reservoir from which aaRS are released when required for their ex-translational functions (31–33).

With this background and these considerations in mind, we were interested to revisit the possible function of the MSC in supporting translation and thus determine the effect on protein synthesis if one or two of its aaRSs were stably and permanently excluded. In contrast to the conflicting earlier studies (19,21,24), which examined protein synthesis broadly on a global scale, we took advantage of more recently developed ribosome-profiling methods to investigate pausing at codons for specific amino acids. Pausing would indicate that the supply of charged tRNAs at the ribosome is insufficient. (We chose codons for arginine and glutamine because of our ability to manipulate those synthetases without disrupting the rest of the complex.) We observed no effects on global protein synthesis and cell growth upon removing ArgRS and glutaminyl-tRNA synthetase (GlnRS) from the MSC, giving MSCΔRQ. These results were unchanged under stress conditions, that is, no differences in translation were observed between cells exposed to inhibitors of central cellular pathways upon expression of the native MSC versus MSCΔRQ. On a molecular level, we could not find differences in ribosome pausing on arginine and glutamine codons between MSCΔRQ-containing and wildtype cells.

Because cue-dependent nuclear activities and mobilizations are increasingly seen as part of the aaRS repertoire of alternative functions (32), we investigated how the presence of ArgRS and GlnRS in the MSC could affect nuclear trafficking of aaRS. This led to the discovery that dislodging ArgRS and GlnRS from the MSC redirected the nuclear distribution of aaRSs, and provided further support for alternative functions, rather than translation *per se*, as the primary role for the MSC.

## MATERIALS AND METHODS

### Cell lines

HEK 293T were cultured in DMEM with 10% serum and penicillin/streptomycin. Immortalized murine embryonic fibroblast cells (MEFs in the following) were a kind gift from Dr Yao Tong in Prof. Xianglei Yang's group at Scripps Research and were cultured in DMEM with 10% serum, pen/strep, β-mercaptoethanol and non-essential amino acids. Cells were regularly tested for mycoplasma using a PCR-based assay and consistently negative.

### CRISPR/Cas9-mediated gene editing

gRNA targeted against exon 2 of ArgRS was designed following rules described by Ran *et al.* (34). pSpCas9(BB)-2A-GFP (PX458) was a gift from Feng Zhang (Addgene plasmid # 48138; http://n2t.net/addgene:48138; RRID:Addgene_48138) (34). Oligos were ordered from Eton Bioscience and inserted into pSpCas9(BB)-2A-GFP to obtain pSpCas9_gRNA_ex2. The plasmid was transfected into HEK 293T cells using Lipofectamine 2000 and immortalized murine embryonic fibroblasts were transfected with a murine version of the gRNA (pSpCas9_gRNA_mex2) using Calfectin. Positive cells were selected by GFP-expression with the help of the Scripps Research Institute Flow cytometry core facility on a MoFlo Astrios EQ jet-in-air sorting flow cytometer (BD Bioscience). Cells were sorted as single cells into each well of a 96-well plate and expanded. The fastest growing 12 clones (HEK 293T) or 17 clones (MEFs) were picked and ArgRS expression was assessed using western blot. 4/12 clones were found with truncated ArgRS corresponding to the expected size, one clone was further found with shorter than full-length ArgRS but not corresponding to the expected molecular weight of ΔLZ-ArgRS in HEK 293T cells. 2/17 clones with truncated ArgRS could be isolated from transfected MEF cells. DNA was isolated from clones with the desired ArgRS variant, the sequence flanking the gRNA-induced cutting site was amplified and sequenced using Sanger sequencing (Eton Bioscience). HEK 293T clone #4 was chosen for its defined frameshift inducing mutation while the exact nature of disruption could not be unambiguously assigned for the other clones. The PCR amplified genetic region flanking the gRNA target site was further subcloned into pET_28b for cleaner sequencing results.

gRNA against human *RARS1* exon 2: attgaccggttgaaaaactg

gRNA against murine *Rars1* exon 2: cttggaagcttctccaagtt

### Western blot

Cells were seeded at a density of 5 × 10^6^ cells on 10 cm dishes or 0.5 × 10^6^ cells/six-well plate. The next day or two days later, protein was collected using 500 (10 cm dishes) or 200 μl (six-well plate) RIPA lysis buffer (from 10x: 0.5 M Tris/HCl, pH = 7.4, 1.5 M NaCl, 2.5% deoxycholate, 10% NP-40, 10 mM EDTA) with protease inhibitor cocktail. Cells underwent a freeze/thaw to ensure lysis and lysate was spun down at 14 000 × g for 15 min. Supernatant was mixed with 5× SDS loading buffer, boiled for 10 min, and 3–5 μl (5–10 μg protein) were loaded on a 4–12% gradient gel. Transfer to PVDF membranes was done using an iBlot or iBlot2. Membranes were blocked in 5% BSA/TBST or 5% milk/TBST for at least 30 min and incubated in the respective antibody diluted in 5% BSA/TBST overnight at 4°C. The following antibodies were used: ArgRS (Biorbyt, 1:5000), ArgRS leucine zipper (Abcam, 1:2000), GlnRS (Proteintech, 1:5000), MetRS (Abcam, 1:3000), GluProRS (Sigma Aldrich, 1:5000), LysRS (Proteintech, 1:1000), TyrRS (made in-house, 1:5000), GlyRS (made in-house, 1:5000), GFP (Proteintech, 1:5000), Puromycin (Millipore, 1:3000), Histone H3 (CST, 1:5000), GAPDH (CST, 1:5000). The next day, membranes were washed 3× in TBST for at least 10 min and incubated for at least 90 min in secondary antibody diluted in 5% BSA/TBST or 2.5% milk/TBST (rabbit secondary: 1:5000, mouse secondary 1:2000, both Invitrogen). Membranes were washed again 3× for at least 10 min. Visualization was done using ECL substrate (Genesee Scientific) on a FluorChem M (Proteinsimple). Western blots were exposed just below saturation to take advantage of the maximum dynamic range. Signal intensities between individual blots are therefore not comparable.

### Co-immunoprecipitation

MetRS interaction partners were detected based on Keilhauser *et al.* (35). 293T wildtype or ΔLZ cells were grown on 15 cm dishes (1 × 10^6^ cells, seeded one day prior) and harvested in 1 ml Tris-buffered saline (TBS) containing 1% IGEPAL CA-630 in TBS for mild lysis. Cells were lysed for 30 min on ice and non-soluble components were pelleted by centrifugation at 14 000 × g for 20 min. Supernatants were loaded on pre-washed Protein A/G agarose beads (SCBT) with 2 μl Anti-MetRS antibody (Abcam). Proteins were enriched for 3 h after which beads were washed twice with 0.1% IGEPAL CA-630 in TBS and twice in TBS only. Beads were stored in PBS at –80°C until further processing. To confirm loss of the N-terminal part of ArgRS, ArgRS was enriched from either wildtype or ΔLZ cell lysate using an anti-ArgRS antibody (Biorbyt).

### Mass spectrometry

MetRS interactomes were eluted from agarose beads by digestion with trypsin in the presence of dithiothreitol (DTT) and iodoacetamide for cysteine alkylation. Digested peptides were desalted using C18 stage tips (Pierce) according to instructions and eluted in 30 μl, yielding ∼1 μg of peptides. The digested samples were analyzed on a Q Exactive mass spectrometer (Thermo). 10 μl of the digest (approximately 250 ng) was injected directly onto a 20 cm, 100 μm ID column packed with Aqua 3 μm C18 resin (Phenomenex). Samples were separated at a flow rate of 400 nl/min on an Easy nLCII (Thermo). Buffer A and B were 0.1% formic acid in 5% acetonitrile and 0.1% formic acid in 80% acetonitrile, respectively. A gradient of 1–35% B over 80 min, an increase to 80% B over 25 min and held at 80% B for 5 min prior to returning to 1% B was used for 120 min total run time. Column was re-equilibrated with buffer A prior to the injection of sample. Peptides were eluted directly from the tip of the column and nanosprayed into the mass spectrometer by application of 2.5 kV voltage at the back of the column. The Q Exactive was operated in a data dependent mode. Full MS^1^ scans were collected in the Orbitrap at 70 K resolution with a mass range of 400–1800 *m*/*z*. The 10 most abundant ions per cycle were selected for MS/MS and dynamic exclusion was used with exclusion duration of 15 s.

Interaction partners were identified as described in Keilhauer *et al.* (35). In brief, peptides were searched and quantified using Maxquant 1.6.7 (36) and further analyzed using Perseus (37). Potential contaminants, peptides only identified by site, or also found in reverse were discarded. LFQ intensities were transformed by log_2_(*x*), and only proteins, which were found in at least two replicates in one group were kept. Missing values were imputed from a normal distribution with a width of 0.3 and a downshift of 1.8. Student's *t*-test was performed to identify significantly enriched proteins.

### Size exclusion chromatography

Cells were seeded on 15 cm dishes to 80–90% confluency. After washing twice with PBS, cells were scraped in 500 μl MSC lysis buffer (20 mM Tris–HCl, pH 7.5, 1 mM EDTA, 150 mM NaCl, 0.1% IGEPAL CA-630 and Protease Inhibitor Cocktail (Pierce)). Cell lysis was completed by incubation on ice for 20 min and soluble proteins obtained by a 10 min spin at 14 000 × g. 500 μl of cell lysate were injected on a Superdex 200 column equilibrated with Dulbecco's phosphate-buffered saline (Gibco). 1 ml fractions were collected, mixed with 5× SDS loading buffer, and 20 μl of each fraction were subsequently analyzed in western blot for the presence of aaRSs.

### Cell fractionation

5 × 10^6^ HEK 293T or MEF cells were seeded on 10 cm dishes one day before harvest. Cells were washed with PBS, scraped in cell fractionation buffer (20 mM HEPES, pH 7.5, 10 mM KCl, 2 mM MgCl_2_, 2 mM EDTA, 1 mM DTT, protease inhibitor), and incubated on ice for 20 min. Cells were passed 10 times through a 27 g needle and centrifuged for 5 min at 750 g to separate nucleus and cytosol. The nuclear pellet was washed three times with cell fractionation buffer and finally solubilized in 2× RIPA at the same volume as the cytosolic fraction. Cytosolic and nuclear fraction were centrifuged at 16 000 × g for 15 min, the supernatant was mixed with 5× SDS loading buffer, and boiled for 10 min. 5 μl of the cytosolic fraction and 10 μl of the nuclear fraction were loaded on a SDS gel and analyzed by western blot analysis.

### Cell viability assay

Cells were seeded at 10 000 cells/well in quadruplets on 96-well plates, a separate plate was used for every time point. 1:10 Alamar blue was added 2 h prior to analysis by fluorescence readout (Synergy H1, Biotek, λ_exc_ = 530 nm, λ_em_ = 580 nm).

### Puromycin incorporation

Cells were seeded on six-well plates one day prior to the experiment at 5 × 10^5^ cells/well. If applicable, small molecule inhibitors were added to the cells for 4 h at the following concentrations: cycloheximide: 50 μg/ml, 17-AAG: 1 μM, Torin: 250 nM, MG132: 10 μM.

For arginine limiting conditions, SILAC DMEM with dialyzed FBS and substituted with lysine was used and arginine was added to the indicated concentration relative to standard DMEM (0.084 g/l). Cells were incubated at the indicated arginine concentration for 6 h. Puromycin was directly added to the medium at 10 μg/ml for 30 min. Afterwards, cells were chased with medium containing the same concentration of inhibitor or arginine for 1 h. Cells were then scraped into lysis buffer (1% Triton X-100, 10 mM β-glycerol phosphate, 10 mM pyrophosphate, 40 mM HEPES pH 7, 2.5 mM MgCl_2_, protease inhibitor) or 1× RIPA and analyzed by western blot analysis.

### Northern blot

Determination of the levels of acylated tRNA *in vivo* was performed as previously described (38). Briefly, total RNA was extracted from cells using Trizol, and the RNA pellet was dissolved in 50 mM sodium acetate (pH 5.0). To deacylate the tRNA, the pellet was dissolved in 0.2 M Tris–HCl, pH 9.5 and incubated for 2 h at 37°C. The RNA was mixed with equal volumes of sample buffer (0.1 M sodium acetate (pH 5.0), 8 M urea, 0.05% bromophenol blue, 0.05% xylene cyanol). 10 μg RNA was separated on a 0.75 mm thick 6.5% polyacrylamide gel containing 8 M urea in 0.1 M sodium acetate buffer (pH 5.0). Electrophoresis was performed in the cold room at 25 mA until the bromophenol blue dye reached the bottom of the gel (∼22 h). The portion of the gel between the xylene cyanol and bromophenol blue bands (which contains the tRNA) was excised and electroblotted onto a Hybond N+ membrane using a Biorad TransBlot cell at 20 V for 90 min in 40 mM Tris–HCl, 2 mM Na_2_EDTA as transfer buffer. The membrane was UV crosslinked, and then hybridized with 5′ end labeled DNA oligonucleotide probes at 55°C in a hybridization buffer containing 6× SSC, 0.01 M sodium phosphate, 1 mM EDTA, 0.25% SDS and 100 μg/ml salmon sperm DNA. Blots were washed beginning with 6× SSC, 0.2% SDS at 55°C and ending with washes in 2× SSC, 0.1% SDS at 65°C.

Probes:

tRNA^Arg^_ACG: GAGCCAGCCAGGAGTCGAACCT
tRNA^Arg^_CCG: CGACCACGAAGGGACTCGAACCCTCA
tRNA^Arg^_TCG: CCGCGGCAGGACTCGAACCTGCAAT
tRNA^Arg^_CCT: TGGGACTCGAACCCACAATCCCTGGCTTAG
tRNA^Arg^_TCT: TAGAAGTCCAATGCGCTATCCATTGCG
tRNA^Gln^_TTG: TCCCACCGAGATTTGAACTCGGA,
tRNA^Gln^_CTG: AGGTTCCACCGAGATTTGAACTCGGA

### Ribosome profiling

Ribosome footprinting was performed as described by Ingolia *et al.* (39). In short, 5 × 10^6^ cells seeded 18 h prior were harvested, RNAseI treated, and polysomes were isolated by ultracentrifugation through a 1 M sucrose gradient at 207 000 × g in a SW55 Ti rotor for 4 h. Replicates represent different passages of the same cell line, with monosomes isolated on consecutive days. Fragments between 26 and 34 nt were isolated, dephosphorylated, and linkers were added. The isolated RNA was then reverse transcribed, circularized and sequences derived from ribosomal RNAs depleted. Finally, fragments were amplified, and barcoding sequences were added. Sequencing was done by the Scripps Next Generation Sequencing Core on a NextSeq500. For details on read depth and mapping, see Supplementary Table S4.

### Identification of pausing sites and observed versus expected

Reads were processed as described in Ingolia *et al.* (39). In brief, adapter sequences were trimmed using Trimgalore and one base on the 5′end was clipped off at the same time (Adapter sequence: CTGTAGGCACCATCAAT). rRNA reads were mapped out using bowtie and remaining reads were aligned against the human transcriptome (v19 release, Genecode) for pausing analysis or against the hg19 human genome (index downloaded from Hisat2) for differential translation analysis, both with Hisat2 (40). Codon usage in the A-site of paused ribosomes were identified as described by Ishimura *et al.* (38) with the following changes: First, bam files were merged and offsets were calculated with functions derived from riboWaltz (41). P-sites were mapped for all reads belonging to transcripts with an average of 0.5 reads/codon, again with functionalities derived from riboWaltz. Finally, pause strength P was calculated as a z-score model assuming a poisson distribution of reads, where *Z* = (read density at codon I – max(background)/(max(background) × 0.5) with a custom python script (Scott Adamson, Jeffrey Chuang (UConn/The Jackson Laboratory), (38)). All codons with *P* ≥ 10 were taken into account. Codons were ranked by how often pausing was observed. For observed/expected, all transcripts with reads were used without minimal criteria for coverage. E-, P- and A-sites were mapped, again with functionalities derived from riboWaltz. The frequency of codons in the A-site was compared to the hypothetical frequency of these codons according to their occurrence in the transcriptome (observed/expected) with a custom python script (Scott Adamson, Jeffrey Chuang (UConn/The Jackson Laboratory), (38)). Differential ribosomal occupancy was calculated using DESeq2 (42). GO analysis was performed using Panther GO with all genes found in ribosome footprints as background (43).

#### Statistical analysis

Student's *t*-test was used to calculate significance (*P < 0.05, **P < 0.01). GraphPad Prism 8 was used for densitometry, RStudio for obs/exp and pauses, Perseus (37) was used for mass spectrometry, and DESeq2 (42) for differential ribosome occupancy. Experiments were performed at different cell passages for repeats.

## RESULTS

### Generation of ArgRS ΔLZ

We deleted a domain of ArgRS that anchors ArgRS to the MSC in mammalian cells (44–46). ArgRS was specifically suitable for this purpose because previous studies identified a full-length and an N-terminally truncated variant that are both derived from the same mature mRNA, with the shorter form utilizing an alternative start codon (47,48). Translation initiation at the second ATG leads to skipping of an evolutionary recent leucine zipper (LZ) at the N-terminus of ArgRS and, while it is still enzymatically active (49,50), the truncated protein is excluded from the MSC.

**Figure 1.**
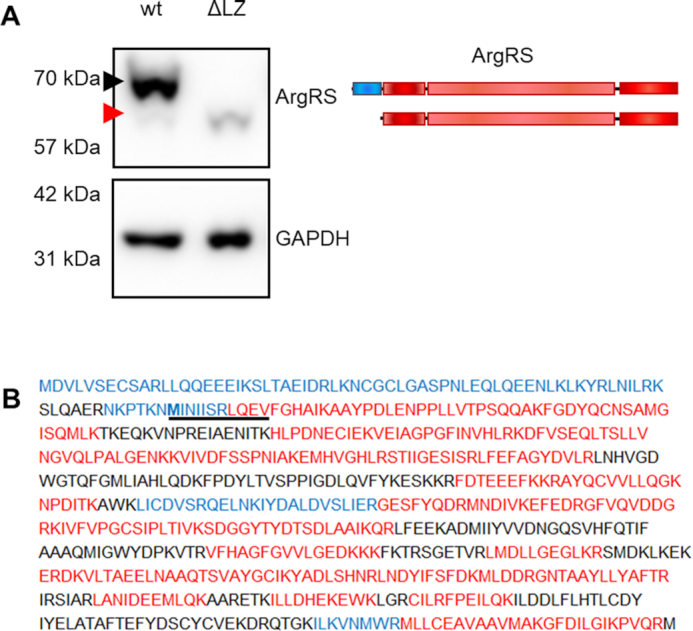
The leucine zipper of genetically encoded arginyl-tRNA synthetase can be skipped by genome editing. (**A**) Full-length ArgRS expression was disrupted by introducing a frameshift in exon 2. ArgRS can subsequently be only expressed as a truncated form lacking the first 1–72 amino acids through initiation from a second ATG start codon (red arrow) as shown by western blot. Black arrow: Full-length ArgRS. A representative out of 5 repeats is shown. Scheme of the ArgRS protein. ArgRS leucine zipper: blue, ArgRS N-terminal, catalytic, and tRNA binding domain: red. (**B**) Peptide coverage following mass spectrometric detection of ArgRS enriched from wild type or gRNA_*RARS1*_ex2 cells confirming the loss of the ArgRS leucine zipper (ΔLZ). Peptides found only in wildtype cells expressing mostly full-length ArgRS are shown in blue, while peptides detected in both or only in ΔLZ cells are red. Black peptides were not detected. Bold: alternative start codon. Coverage was mapped from three repeats.

We investigated whether the remaining aaRSs in the MSC can preserve their interactions following permanent exclusion of ArgRS from the MSC. We could confirm endogenous expression of ArgRS variants corresponding to both expected molecular weights in all tested cell lines (Supplementary Figure S1A). (This shorter form of ArgRS will subsequently be referred to as ΔLZ.) In order to understand the difference between free ΔLZ ArgRS and MSC-bound full-length ArgRS, we developed a cell line that only expresses ΔLZ ArgRS. We generated stable HEK 293T cell lines with CRISPR/Cas9 that are disrupted in their endogenous ArgRS gene *RARS1* in exon 2 (Supplementary Figure S1B–D). Translation from the first ATG start codon led to a premature stop codon. However, ArgRS would still be translated starting from the second ATG, thus supplying sufficient active ArgRS to support cells (Figure 1A). Expression of the ΔLZ form of ArgRS doubled in comparison to that seen in wildtype cells while total full-length and ΔLZ ArgRS expression was reduced to 10% (Figure 1A, Supplementary Figure S1E). Lack of full length ArgRS was verified by mass spectrometry (Figure 1B, Supplementary Table S1).

### Loss of ArgRS from the MSC dislocates GlnRS but not other aaRSs

**Figure 2.**
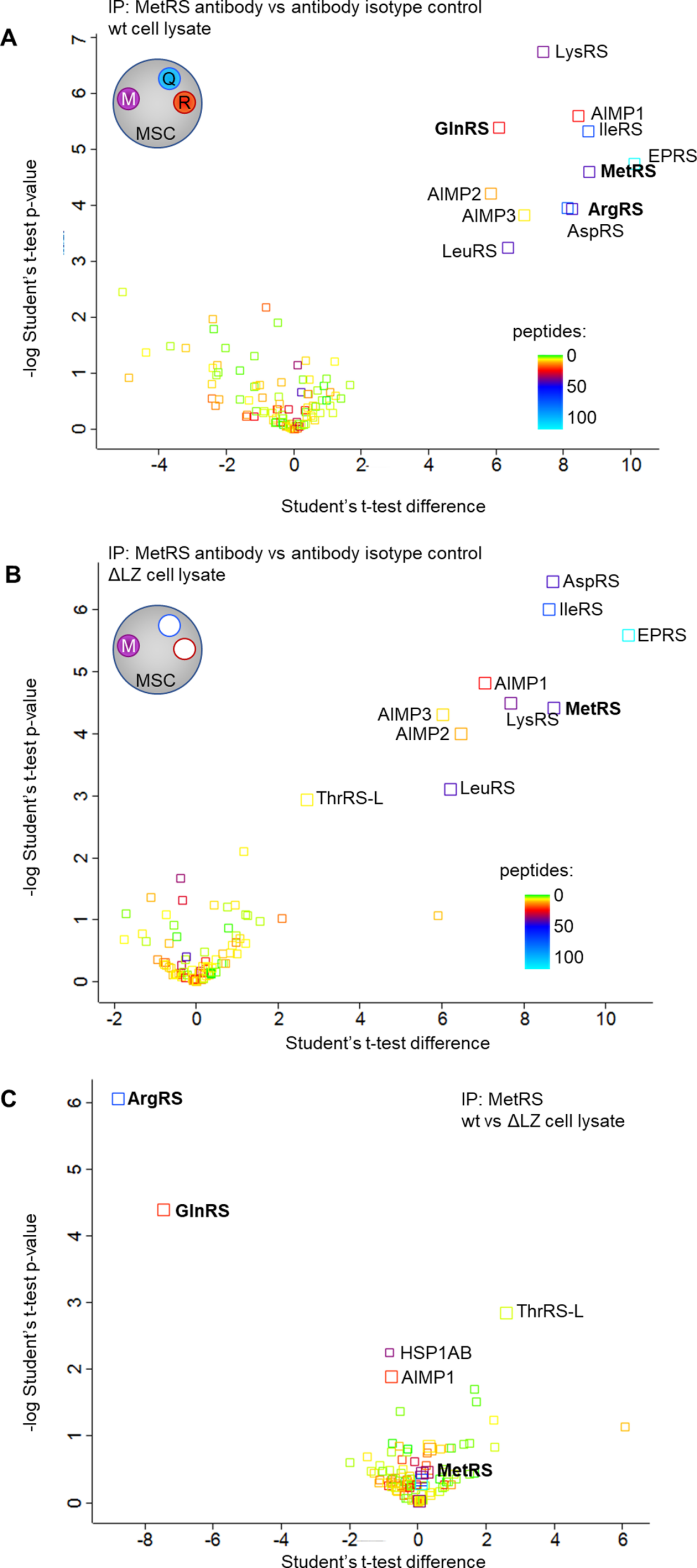
Deletion of the ArgRS LZ excludes ArgRS and GlnRS from the MSC. (A-C) Volcano plot of proteins identified by mass spectrometry following co-immunoprecipitation of MetRS interaction partners (3 repeats). The number of identified unique and razor peptides is visualized by color. Interaction partners of methionyl-tRNA synthetase (MetRS), a protein in the multisynthetase complex, in HEK 293T (**A**) wildtype and (**B**) ΔLZ cells. (**C**) Wildtype vs ΔLZ cells: ArgRS and GlnRS are lost from the MetRS interactome upon loss of the ArgRS LZ.

Previous work showed that the LZ of ArgRS interacts with AIMP-1 to anchor ArgRS in the MSC (46). MSC integrity was probed using two distinct techniques: First, we assessed the interactome of another MSC-bound aaRS, methionyl-tRNA synthetase (MetRS, MARS1), using immunoprecipitations (IP) from wildtype and ΔLZ cells followed by mass spectrometry (Figure 2A–C). We chose MetRS to assess MSC integrity because it is located in a different MSC subcomplex and no direct interaction between ArgRS and MetRS has been previously reported (10,51). In wildtype 293T cells, the MetRS interactome was restricted to the MSC components and all 8 aaRSs and three adaptor proteins could be retrieved (Figure 2A, Supplementary Table S2). In ΔLZ cells, ArgRS was not found in the MetRS interactome, verifying that the presence of ArgRS in the MSC is dependent on its LZ domain. In addition to ArgRS, GlnRS (QARS1), was also lost from the MetRS interactome, supporting previous reports (46) that ArgRS anchors GlnRS to the MSC, *via* the ArgRS LZ (Figure 2B, C, Supplementary Table S3).

**Figure 3.**
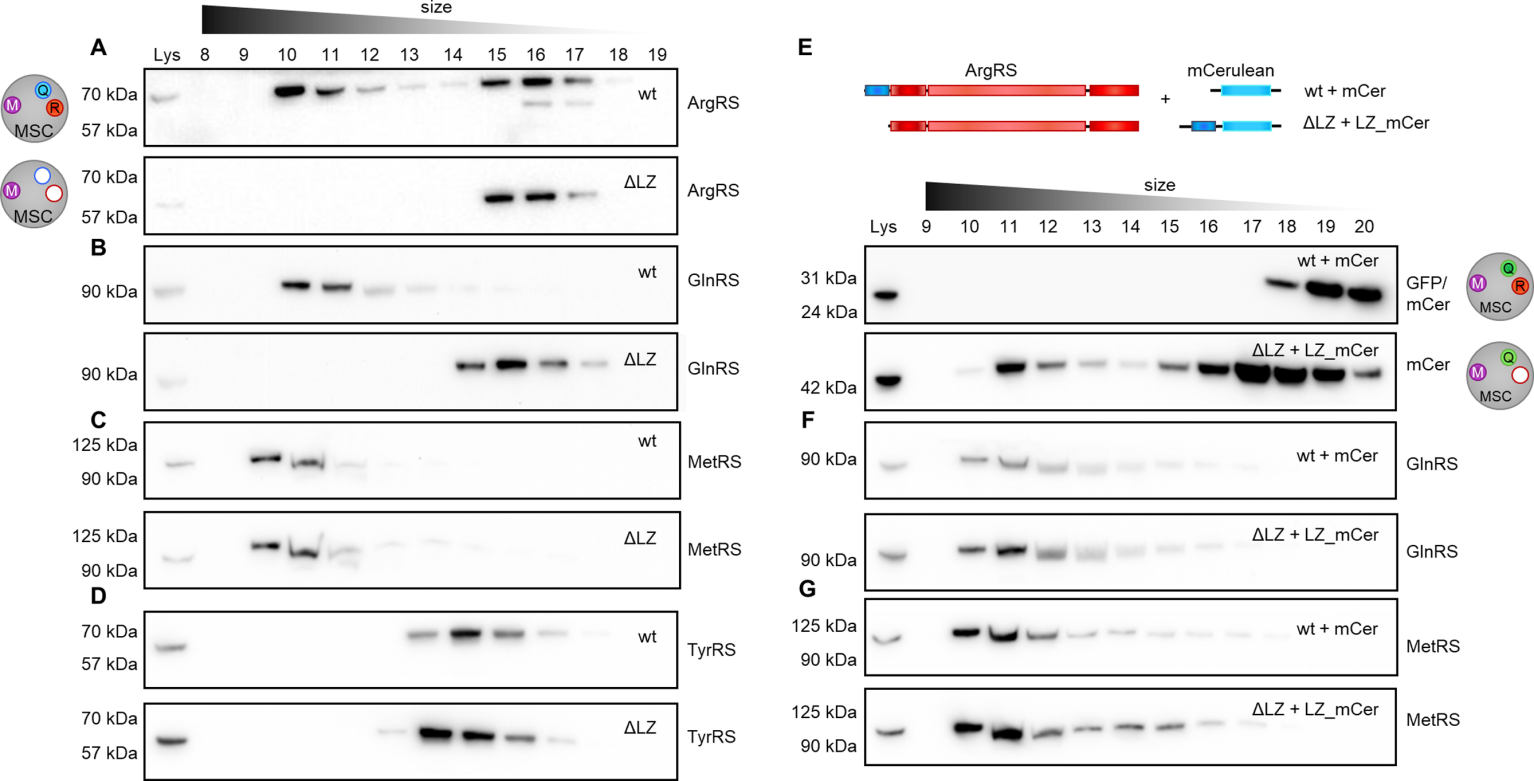
The MSC persists as a macromolecular complex in the absence of ArgRS and GlnRS. Cell lysate was separated by size exclusion chromatography to distinguish between monomeric/dimeric and multisynthetase complex-bound proteins. aaRSs were identified by western blot. Intact multisynthetase complex elutes between fraction 10–12. Dimeric or monomeric tRNA synthetases elude between fraction 14–17. (A–D) wt: wildtype 293T cells, ΔLZ: 293T cells expressing only the truncated version of ArgRS lacking the N-terminal leucine zipper. (**A**) Arginyl-tRNA synthetase, ArgRS. Exposure times differ to accommodate for lower expression levels of ArgRS ΔLZ. (**B**) Glutaminyl-tRNA synthetase, GlnRS. (**C**) Methionyl-tRNA synthetase, MetRS. (**D**) Tyrosyl-tRNA synthetase, TyrRS. (E–G) The leucine zipper of ArgRS was fused to the N-terminus of mCerulean (LZ-mCer). Monomeric mCerulean elutes between fraction 17–18. mCer: mCerulean, cyan fluorescent protein. ΔLZ_mCer: mCerulean with the leucine zipper of ArgRS fused to its N-terminus. Scheme of the LZ-mCerulean fusion protein and ArgRS variant. ArgRS LZ: blue, ArgRS N-terminal, catalytic and tRNA binding domain: red. mCerulean: light blue. (**E**) mCerulean (detected by an anti-GFP antibody). (**F**) Glutaminyl-tRNA synthetase, GlnRS. (**G**) Methionyl-tRNA synthetase, MetRS.

To confirm that the MSC components were indeed assembled in one large complex as opposed to disintegrating into smaller subcomplexes, a second method was used. Under mild conditions the MSC can be retrieved intact from cell lysates (52). After separation of wildtype cell lysate with a size-exclusion column (SEC), the majority of MSC-bound aaRSs, including ArgRS, GlnRS and MetRS, appeared in fractions 10–12 (Figure 3A–C). Free or dimeric aaRSs (as exemplified by tyrosyl-tRNA synthetase (TyrRS), a dimer that is not a part of the MSC, Figure 3D) eluted at fractions 12–16, in line with their lower molecular weight, and could thus be separated from MSC-bound aaRSs. In ΔLZ cells, ArgRS and GlnRS eluted in later fractions (15–17 and 14–17, respectively, Figure 3A, B), while MetRS continued to elute in fraction 10–12, in a profile comparable to wildtype cells (Figure 3C). This finding was in accordance with the characterization of the MetRS interactome (Figure 2). Interestingly, 40% of ArgRS in wildtype cells was not bound to the MSC (Supplementary Figure S2A), while free MetRS and GlnRS were barely detectable (Figure 3B, C). In summary, deletion of the ArgRS LZ displaced ArgRS itself and GlnRS from the MSC but did not disrupt the remaining synthetases nor the overall integrity of the complex.

### The ArgRS leucine zipper is sufficient to rescue the loss of GlnRS from the MSC

As deletion of the LZ from ArgRS excluded GlnRS from the MSC, we hypothesized that expressing the ArgRS LZ would be sufficient to rescue the loss of GlnRS from the MSC. We therefore fused the ArgRS LZ to the N-terminus of cyan fluorescent protein mCerulean (subsequently abbreviated as LZ_mCer, Supplementary Figure S2B). SEC showed that LZ_mCer eluted with the MSC while unmodified mCerulean eluted at a later fraction (Figure 3E). In living cells anchoring of GlnRS to the MSC is dependent on the ArgRS LZ-domain, as the GlnRS elution profile shifted back to higher molecular weight fractions upon expression of LZ_mCer (Figure 3F). Notably, elution profiles of ArgRS or other MSC-bound aaRSs were not affected by the introduction of LZ_mCer (Figure 3G, Supplementary Figure S2C). This suggests that LZ_mCer substituted for ArgRS in the MSC without affecting other aaRSs and resulted in the formation of an MSC only lacking ArgRS (MSCΔR).

### tRNA^Arg^ and tRNA^Gln^ are aminoacylated despite the loss of ArgRS and GlnRS from the MSC

**Figure 4.**
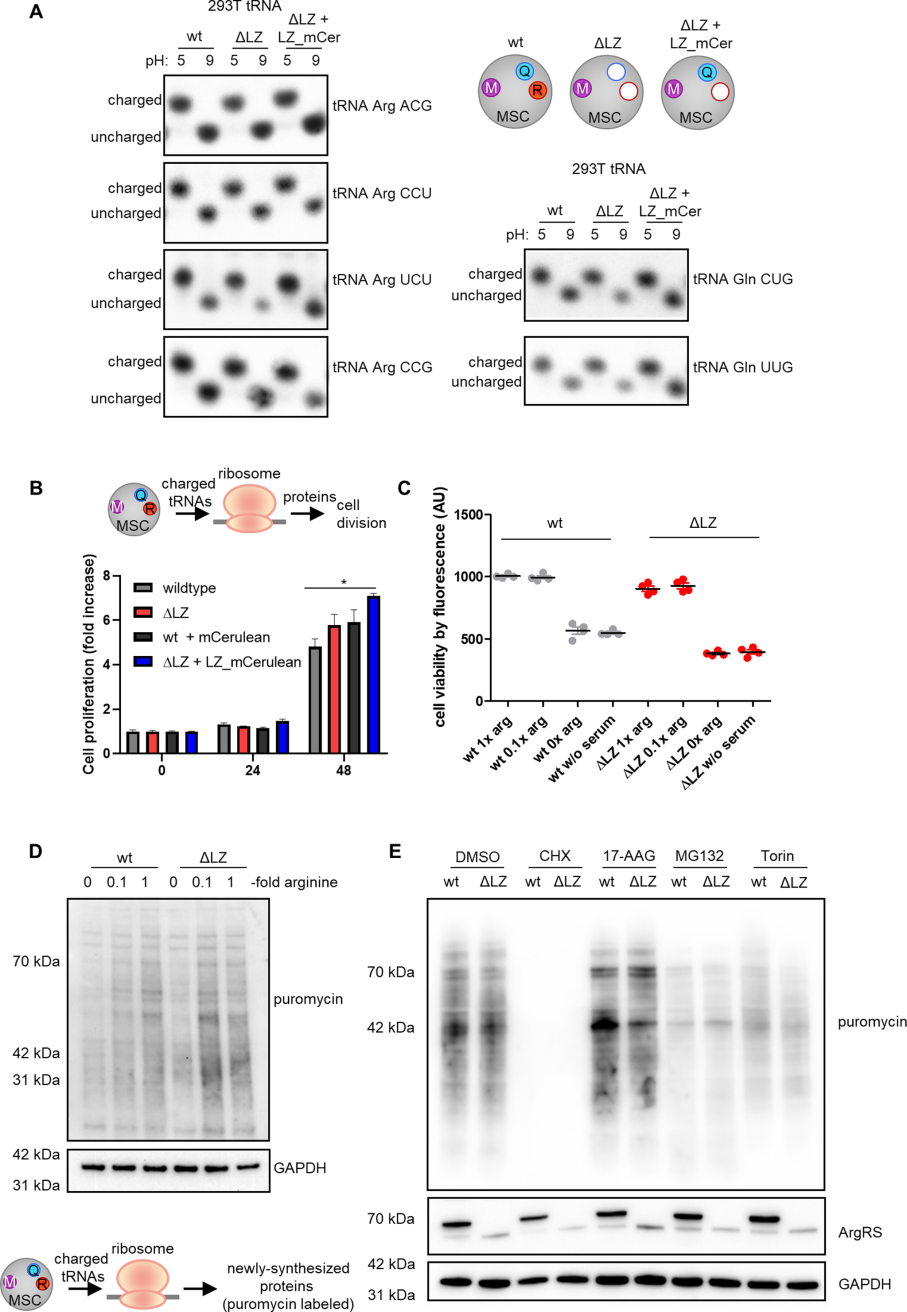
Loss of ArgRS and GlnRS from the multisynthetase complex does not affect tRNA charging, mRNA translation, or cell viability. (A–E) wt: wildtype 293T cells, ΔLZ: 293T cells expressing only the truncated version of ArgRS lacking amino acid 1–72. LZ-mCer: the ArgRS leucine zipper was fused to mCerulean and rescues the loss of GlnRS from the MSC. (**A**) Charged versus uncharged Arg- and Gln-tRNA levels by northern blot (three repeats). The aminoacylation state of the tRNA can be preserved by acidic conditions (pH 5), while the aminoacyl-tRNA-bond hydrolyzes at neutral or basic pH (pH 9). (**B**) Cell viability on different days after seeding was assessed by Alamar Blue. Mean of quadruplets are shown with standard derivation. A representative out of three repeats is shown. (**C**) Alamar blue cell viability assay in different arginine concentrations after 24 h. Experiments were performed in technical quadruplets and a representative out of three repeats is shown. (**D**) Western blot detection of puromycin incorporation to visualize newly synthesized proteins. Arginine-fold changes are relative to DMEM arginine levels. A representative out of four repeats is shown. (**E**) Western blot detection of puromycin incorporation under cell stress. CHX: cycloheximide, ribosome inhibitor. MG132: proteasome inhibitor. Torin: mTORC1 inhibitor. 17-AAG: Hsp90 inhibitor. A representative out of four repeats is shown.

A previous study describing a high-throughput sequencing method showed that tRNAs^Arg^ and tRNAs^Gln^ are >90% charged (53). To directly assess whether the exclusion of ArgRS would influence the aminoacylation status of tRNA^Arg^ or tRNA^Gln^ in cells, we used northern blots to visualize the percentage of tRNA that is charged. If tRNA is isolated in acidic conditions, the amino acid remains attached; charged and uncharged tRNA can then be separated by their size. No bands corresponding to uncharged tRNA^Arg^ and tRNA^Gln^ (Figure 4A) were found even when their respective aaRSs were dislodged from the MSC. Thus, both tRNAs were completely aminoacylated, suggesting that ArgRS and GlnRS access their substrates irrespective of whether they are bound to the MSC.

### Cell proliferation and viability are unchanged upon loss of ArgRS and GlnRS from the MSC

As the multisynthetase complex harbors nine catalytic activities for the charging of tRNAs with their cognate amino acids, it is intuitive to assume that complex formation is beneficial for protein synthesis. The conservation of the MSC between species and the early occurrence of aaRS complexes in evolution further suggest an advantage in clustering aaRSs together (16,17). In contrast to previous findings (21), cell viability and proliferation in 293T cells harboring MSCΔRQ or MSCΔR were comparable to wildtype cells (Figure 4B). Arginine is a conditionally essential amino acid (54) and HEK cells are dependent on arginine from their cell culture medium (55). With this in mind, we limited available arginine. Cell viability was unchanged in wildtype versus ΔLZ cells, even with low arginine concentrations (Figure 4C).

### Protein synthesis is unaffected by loss of ArgRS and GlnRS from the MSC

To investigate further whether exclusion of ArgRS and GlnRS from the MSC hindered mRNA translation, we assayed the ability of ΔLZ cells to synthesize proteins. Previously, CHO cells harboring ΔLZ ArgRS and a heat-sensitive, full-length mutant of ArgRS were reported to be deficient in protein synthesis despite comparable levels of charged tRNA (21). We revisited these findings by exposing cells to puromycin to label newly synthesized proteins (56). As expected, arginine starvation reduced puromycin incorporation and confirmed that limiting arginine attenuates protein biosynthesis (Figure 4D, Supplementary Figure S2D). However, despite a slight reduction at 0.1× arginine (Supplementary Figure S2D), newly synthesized proteins were not reduced at standard arginine levels in ΔLZ cells lacking MSC bound ArgRS and GlnRS. This confirmed that ribosomes in ΔLZ cells are provided with sufficient tRNA^Arg^ and tRNA^Gln^ for protein synthesis.

Stress conditions can elevate the need for efficient cell metabolism. To induce stress, we exposed wildtype and ΔLZ cells to small molecule inhibitors of different cellular pathways. As expected, inhibition of translation elongation by cycloheximide (CHX) (56) led to a strong reduction of protein synthesis (Figure 4E, Supplementary Figure S2D). HSP90 inhibition by 17-AAG (which can result in a heat shock-like response (57)), proteasome inhibition with MG132 (which impairs degradation of ubiquitinated proteins (58)), and mTORC-1 inhibition by Torin-1 (59), did not lead to differences in puromycin labelling between wildtype and ΔLZ cells (Figure 4E, Supplementary Figure S2D). Thus, exclusion of ArgRS and GlnRS from the MSC does not impact protein synthesis within the sensitivity detectable by puromycin incorporation.

### No pausing or increased ribosome occupancy observed at arginine or glutamine codons upon exclusion of ArgRS and GlnRS from the MSC

Puromycin incorporation provides a snapshot of ongoing translation during the pulse-period (56). However, it does not offer codon-level resolution. Previous work demonstrated that arginine limitation leads to pausing on arginine codons, which could be detected in ribosome footprints (60). In addition, another study suggested disruption of tRNA channeling to the ribosome upon exclusion of an aaRS from the MSC (21). Therefore, a decrease in efficiency due to disruption of tRNA channeling by displacing ArgRS and GlnRS from the MSC would cause ribosome slowdown specifically at the aaRSs’ cognate codons, which would be evident as increased ribosomal density. For codon-level resolution of ongoing translation, we therefore performed ribosome footprint analysis on wildtype, ΔLZ, and ΔLZ + LZ_mCer cells (Supplementary Table S4). We restrained from limiting external arginine as it leads to strong pausing and inactivates the translation-regulating mTOR pathway (60). Such limiting could also mask the more nuanced effects we expect based on puromycin incorporation (Figure 4).

To study whether mRNA translation was hindered at arginine or glutamine codons in MSCΔRQ or MSCΔR-carrying cells, we mapped ribosomal E, P, and A-sites to the acquired ribosome footprints. An increase in ribosomes with Arg or Gln codons in the A-site over the expected frequency calculated from mRNA sequences would indicate stalling at these codons (61,38). In addition, we also counted how often pausing (defined by a pause score of *P* ≥ 10 over background (38)) occurred on Arg and Gln codons in wildtype, ΔLZ, and ΔLZ + LZ_mCer.

**Figure 5.**
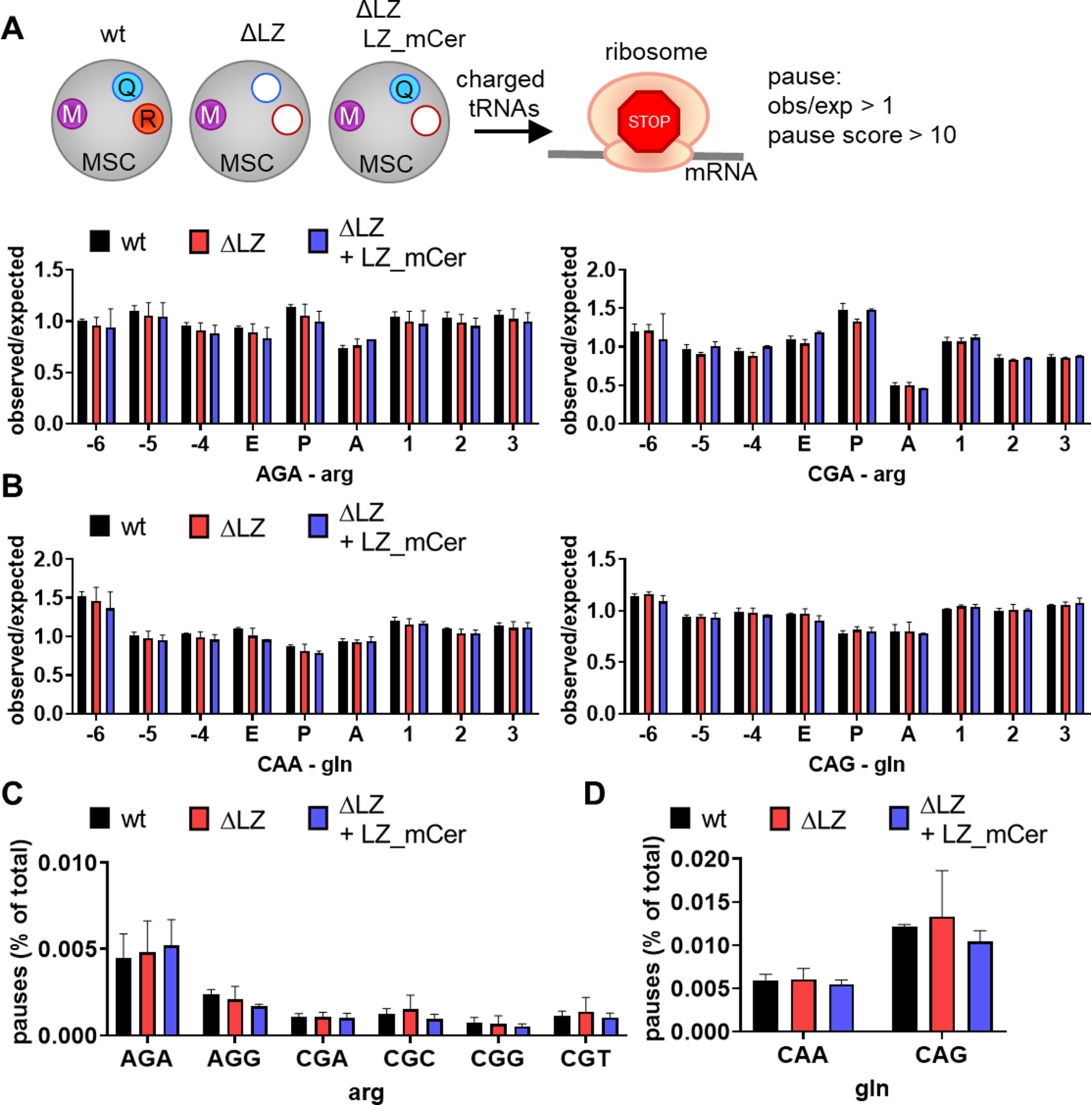
No indication for ribosome stalling on cognate codons upon loss of ArgRS and/or GlnRS from the MSC. (A–D) Analysis of ribosome footprints for pausing on arginine or glutamine codons. wt: wildtype 293T cells, ΔLZ: 293T cells expressing only the truncated version of ArgRS lacking the N-terminal leucine zipper (LZ), LZ_mCer: ArgRS LZ fused to mCerulean. Three repeats. (A, B) Observed ribosome occupancy versus expected occupancy based on genetic codon usage in the E, P, and A-site of the ribosome in arginine (**A**) and glutamine (**B**) codons calculated from ribosome footprints. (C, D) Percentage of arginine (**C**) and glutamine (**D**) codons among all codons with a pause score *P* ≥ 10 calculated from ribosome footprints.

Neither a higher observed/expected occupancy at Gln or Arg codons in the A-site of ribosomes (Figure 5A, B, Supplementary Figure S3A), nor increased pausing on Arg or Gln codons was found (Figure 5C, D, Supplementary Figure S3B). We then analyzed differential ribosomal occupancy to assess whether loss of ArgRS and/or GlnRS from the MSC would affect translation of specific genes. Using a stringent cutoff (padj<0.05, Δ|log2foldchange| > 1.5) less than 20 out of 16266 genes displayed differential ribosomal occupancy in ΔLZ or ΔLZ + LZ_mCer versus wildtype (Supplementary Figure S3C, Supplementary Tables S5 and S6). Most of these were shared between ΔLZ and ΔLZ + LZ_mCer, suggesting that either the reduction of ArgRS or its displacement from the MSC led to the majority of differentially translated genes as opposed to the loss of GlnRS from the MSC. Without a fold-change cutoff (*P*_adj_ < 0.05, 341 genes in ΔLZ versus wildtype) genes involved in developmental pathways were enriched, especially those associated with pattern formation and neurodevelopment (Supplementary Figure S3D).

### ArgRS exclusion from the MSC does not hinder murine cell growth

In addition to human HEK 293T cells, we also generated immortalized mouse embryonic fibroblast (MEF) cells lacking full-length ArgRS (Supplementary Figure S4A, B). ArgRS was excluded from the MSC (Supplementary Figure S4C) in these MEF cells. In line with results obtained in human cells, proliferation and protein synthesis were not reduced, even under arginine limited conditions (Supplementary Figure S4D, E). Thus, this dispensability of MSC-bound ArgRS is not restricted to HEK 293T cells and is shared between species.

### Exclusion of ArgRS and GlnRS from the multisynthetase complex leads to reduction of nuclear MSC aaRSs

**Figure 6.**
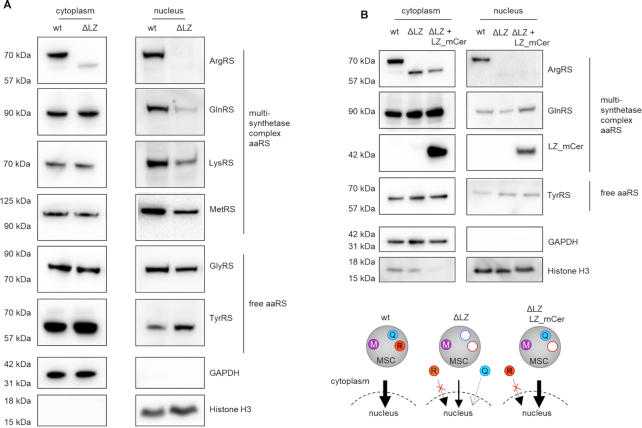
Loss of ArgRS leucine zipper lowers nuclear levels of MSC aaRSs. (A, B) Arginyl-tRNA synthetase, ArgRS. Glutaminyl-tRNA synthetase, GlnRS. Lysyl-tRNA synthetase, LysRS. Methionyl-tRNA synthetase, MetRS. Glycyl-tRNA synthetase, GlyRS. Tyrosyl-tRNA synthetase, TyrRS. GAPDH: cytoplasm loading control. Histone H3: nuclear loading control. Western blots were imaged with different exposures for cytoplasmic and nuclear fractions. (**A**) Western blot of cytoplasmic and nuclear fraction from wildtype cells and cells with truncated ArgRS (ΔLZ). (**B**) Western blot of the cytoplasmic and nuclear fraction from wildtype cells, cells with truncated ArgRS (ΔLZ), and ΔLZ cells expressing ΔLZ_mCer.

Our findings suggest that the enzymatic function of an aaRS is not altered by its presence or absence from the MSC. We therefore investigated alternative functions of the MSC and asked whether the MSC determines cellular localization of its aaRSs. MSC-bound aaRSs have been previously found in the nucleus (62) where they contribute to tRNA quality control (63) as well as ex-translational functions (64). We probed the cellular localization of MSC aaRSs by comparing the levels of MSC components in the cell nucleus of wildtype (MSC) versus ΔLZ cells (MSCΔRQ). Six percent of ArgRS was found in the nuclear fraction (Supplementary Figure S5A). Only full-length ArgRS appeared in the nucleus and loss of the leucine zipper excluded it from the nucleus (Figure 6A). GlnRS nuclear levels in ΔLZ cells were reduced to 30% in comparison to wildtype (Figure 6A, Supplementary Figure S5B), while cytoplasmic GlnRS levels did not change (Supplementary Figure S5B). Because 90% of GlnRS was found in the cytoplasm (Supplementary Figure S5A), it can be assumed that the amount of total GlnRS was comparable between wt and ΔLZ cells. In addition to ArgRS and GlnRS, other tested MSC-bound aaRSs were also reduced in the nucleus of ΔLZ cells as compared to wildtype (Figure 6A, Supplementary Figure S5B). Cytosolic levels of aaRSs were unchanged for both MSC-bound and free aaRSs. The severe reduction of ArgRS and GlnRS in the nuclear fraction of ΔLZ cells suggests that their presence in the MSC is a prerequisite for their nuclear localization (Figure 6A). Importantly, reintegration of GlnRS in the MSC by LZ_mCer rescued nuclear levels of GlnRS in comparison to ΔLZ cells and restored nuclear levels of other tested MSC aaRSs (Figure 6B, Supplementary Figure S5B). Thus, ArgRS with its naturally fused LZ is itself a regulator of the cellular distribution of MSC components.

## DISCUSSION

While important progress has been made on the structure of the MSC, it is still not known in full molecular detail (51,65,66). We show here that arginyl-tRNA synthetase can be excluded from the MSC by ablation of its N-terminal leucine zipper (LZ) (Figures 2 and 3). Due to the interaction between the leucine zipper of ArgRS, AIMP1, and GlnRS, exclusion of ArgRS also removed GlnRS from the MSC (Figures 2 and 3A-D, MSCΔRQ). Expression of the ArgRS LZ was sufficient to reintroduce GlnRS to the MSC (Figure 3E–G, MSCΔR). Consistent with an ex-translational role for the MSC, the MSCΔRQ had no adverse effects on cell proliferation, tRNA charging, mRNA decoding, or protein synthesis (Figures 4 and 5). Interestingly, the nuclear presence of ArgRS is dependent on its N-terminal LZ and, further, loss of full-length ArgRS reduced other MSC-bound aaRSs in the nuclear fraction (Figure 6).

Previous work suggested that the MSC contributes to protein synthesis by channeling tRNAs to the ribosome (21). In the earlier work (21), mutant, temperature-sensitive endogenous ArgRS was inactivated in CHO cells by heat and cells were rescued by ectopically expressing ArgRS variants (21). Mutating the first start codon in ectopically expressed ArgRS led to reduced protein synthesis and a growth defect despite comparable levels of charged tRNA^Arg^ (21). Here, we directly manipulated the endogenous *RARS1* gene to force expression of ΔLZ only, and thereby avoided the need for continuous heat exposure as well as interference from residual, inactive mutant ArgRS. In contrast to previous work, neither on a proteome-wide nor on a specific-codon basis could we find evidence for disrupted protein synthesis upon the exclusion of ArgRS and GlnRS (Figures 4 and 5). Finer differences at the level of single codons might become more visible if arginine would be limited. Our study does not rule out the concept of tRNA channeling to the ribosome, which has been described for both MSC-bound and free aaRSs (67). Rather it shows that, if channeling of tRNA^Arg^ and tRNA^Gln^ from the MSC takes place, it is either dispensable in dividing cell lines or the localization of ArgRS and GlnRS in the MSC is not a pre-requisite for channeling. The latter is in line with previous reports in which channeling was shown for both dimeric, free-standing aaRS and a complex-bound aaRS (67). In addition, deletion of the adapter protein AIMP-2, which led to the complete disassembly of the MSC, did not affect global protein synthesis (19,24).

While the absence of ArgRS and GlnRS from the MSC did not impact translation of most mRNAs, genes associated with development, for example homeobox and forkhead box genes, were differentially translated in MSCΔRQ cells (Supplementary Figure S3D). Translation of these genes is spatially regulated in development and aaRSs might be localized by the MSC for specialized or localized translation needs. It is noteworthy that, despite comparable levels of aminoacylation activity, a patient with a mutation resulting in the predominant expression of ΔLZ ArgRS displayed clinical symptoms (68). These findings, when viewed in the light of our data, raise the possibility that patient cells have experienced a redistribution of MSC components that introduces or disrupts ex-translational functions or specialized translation in an unregulated way.

In contrast to the lack of effect on general mRNA translation, we found that exclusion of ArgRS from the MSC changed the cellular distribution of MSC components. It is unclear how the ArgRS LZ domain and GlnRS contribute to the nuclear distribution of the MSC, as neither contain nuclear localization sequences (NLS). Despite significant advances in our understanding of the MSC structure (51,65,66), it is yet unclear whether arrangements in the MSC could control exposure of other aaRS’s nuclear import or export cues upon ArgRS exclusion.

Several studies showed that mobilization of aaRSs from the MSC occurs upon a specific stimulus. For example, GluProRS is phosphorylated when mTOR and CDK5 are activated (28,69), and this cue leads to new signaling events. LysRS is excluded from the MSC upon phosphorylation at its binding interface with an MSC scaffold protein and this in turn leads to a role in transcriptional control (29,30). Cue-dependent ex-translational activities for other MSC-bound aaRSs have also been described, including GlnRS, MetRS, and LeuRS (70–73). Because MSC aaRSs fulfill their ex-translational functions through interactions with non-MSC factors, and because these interactions might be sterically hindered, release of an aaRS from the MSC might offer an additional step of control over signaling activities. While the fate of the remaining MSC proteins is unclear, in the specific case we report here, loss of individual aaRSs did not force disassembly of the MSC (Figures 2 and 3).

Related to this concept, most Charcot-Marie-Tooth Disease causing mutations in tRNA synthetases are in free-standing, non-MSC bound aaRSs (9). Moreover, several pathologic aaRS mutations cause aberrant synthetase interactions due to conformational openings of the aaRS that expose previously hidden protein surfaces (9). Thus, a possible evolutionary force for sequestration of aaRSs in the MSC is the restraint it imposes on aberrant interactions.

## DATA AVAILABILITY

Ribosomal footprinting data is deposited at GEO with the accession number GSE158212. The mass spectrometry proteomics data have been deposited to the ProteomeXchange Consortium via the PRIDE partner repository with the dataset identifier PXD021527 and 10.6019/PXD021527.

## Supplementary Material

gkaa1183_Supplemental_Files
