# Supplementary material for: Regulation of ex-translational activities is the primary function of the multi-tRNA synthetase complex
Source: Nucleic Acids Res. 2020 Dec 21;49(7):3603–16. doi: 10.1093/nar/gkaa1183 (PMC8053116; doi:10.1093/nar/gkaa1183)
Supplement: gkaa1183_Supplemental_Files [file gkaa1183_supplemental_files.zip › 201116_RARS_ex2_Supp.pdf]

Supplementary Figure 1

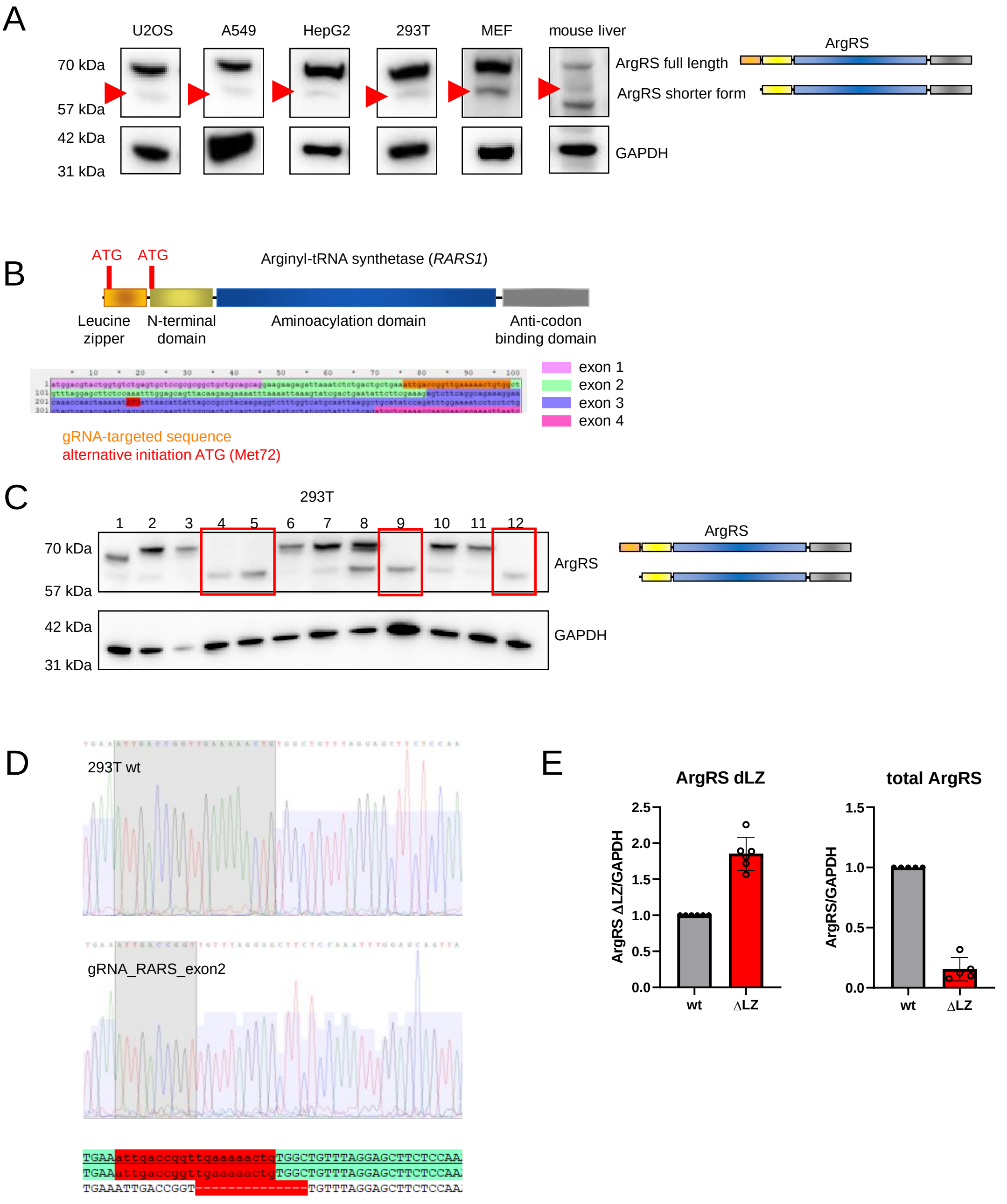

Supplementary Figure 1: Endogenous and forced expression of a truncated form of ArgRS. (A) Western blot of endogenous ArgRS showed expression of two ArgRS variants in different cell lines and species. U2OS: human bone osteosarcoma, A549: human lung carcinoma, HepG2: human hepatocellular carcinoma, 293T: human embryonic kidney cells, MEF: immortalized murine embryonic fibroblasts, mouse liver: C57Bl/6 liver. The size of the lower band corresponds to the expected size of ArgRS starting from an alternative start codon (red arrows). (B) Scheme of a CRISPR/Cas9-based approach to disrupt full-length ArgRS expression. The sequence targeted by the gRNA lies in exon 2 of the ArgRS gene *RARS1*. (C) Western blot of ArgRS in cell lysate of different HEK293T clones. The fastest growing 12 clones after single cell sorting were analyzed. The desired edit of the ArgRS gene *RARS1* was indicated by loss of the full-length ArgRS band (red box). Partial edits, resulting to heterozygous cells or incomplete truncations were also observed (clone 1, 8). Clone 4 was used for the remaining studies. (D) Sanger sequencing of the RARS1 gene after editing and in wildtype. Induction of a frameshift leads to the insertion of a stop codon 33 amino acids after the first initiation codon and termination of the full-length protein. The shadowed area is complementary to the gRNA. (E) Quantification of ArgRS by densitometric analysis of Western blots (representative shown in Figure 1B).

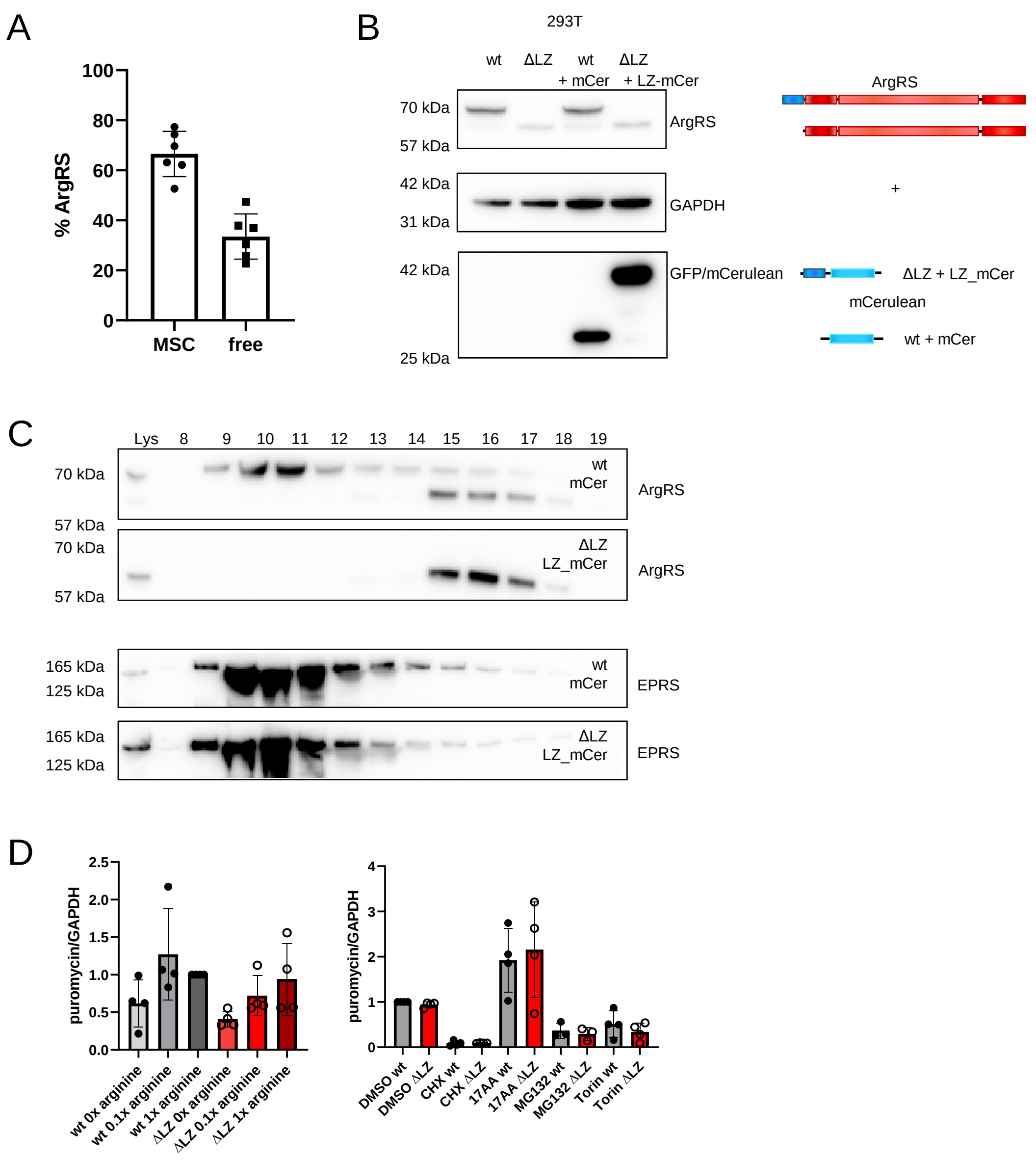

Supplementary Figure 2: Percentage of MSC-bound ArgRS, expression of LZ_mCerulean, and quantification of puromycin incorporation. (A) Percentage of MSC-bound ArgRS (fractions 10-12 in Figure 3A) and free ArgRS (fraction 15-17 in Figure 3A), quantified by densitometry after size exclusion chromatography of HEK 293T wildtype cell lysates and Western blot. (B) Detection of stable expression of unmodified or LZ-fused mCerulean in HEK 293T wildtype or ΔLZ cells by Western blot. The leucine zipper of ArgRS (amino acids 1-72) was fused to the N-terminus of mCerulean (LZ-mCer). Scheme of the LZ-mCerulean fusion protein and ArgRS variant. ArgRS LZ: blue, ArgRS N-terminal, catalytic, and tRNA binding domain: red. mCerulean: light blue. (C) Size exclusion chromatography of cell lysate followed by aaRSs detection by Western blot. Intact multisynthetase complex eluted between fraction 10-12. Dimeric or monomeric tRNA synthetases eluted between fraction 14-17. ArgRS was excluded from the MSC in ΔLZ cells and was not relocated to the MSC upon expression of LZ_mCer. The presence of EPRS in earlier fractions suggested that the remaining MSC was intact in ΔLZ + LZ_mCer cells. (D) Densitometric quantification of puromycin incorporation as quantified from Western blots (representative shown in 4D, E). No significant differences were found in left panel except for wt 0.1x arginine vs ΔLZ 0x arginine, $p=0.03$. In the right panel, no significant differences were found between wt and ΔLZ exposed to the same inhibitors. Treatment with all inhibitors except 17AA led to significant differences to the DMSO control. ΔLZ DMSO vs ΔLZ CHX: $p< 0.001$, ΔLZ DMSO vs ΔLZ 17AA: n.s., ΔLZ DMSO vs ΔLZ MG132: $p=0.003$, ΔLZ DMSO vs ΔLZ Torin: $p=0.001$.

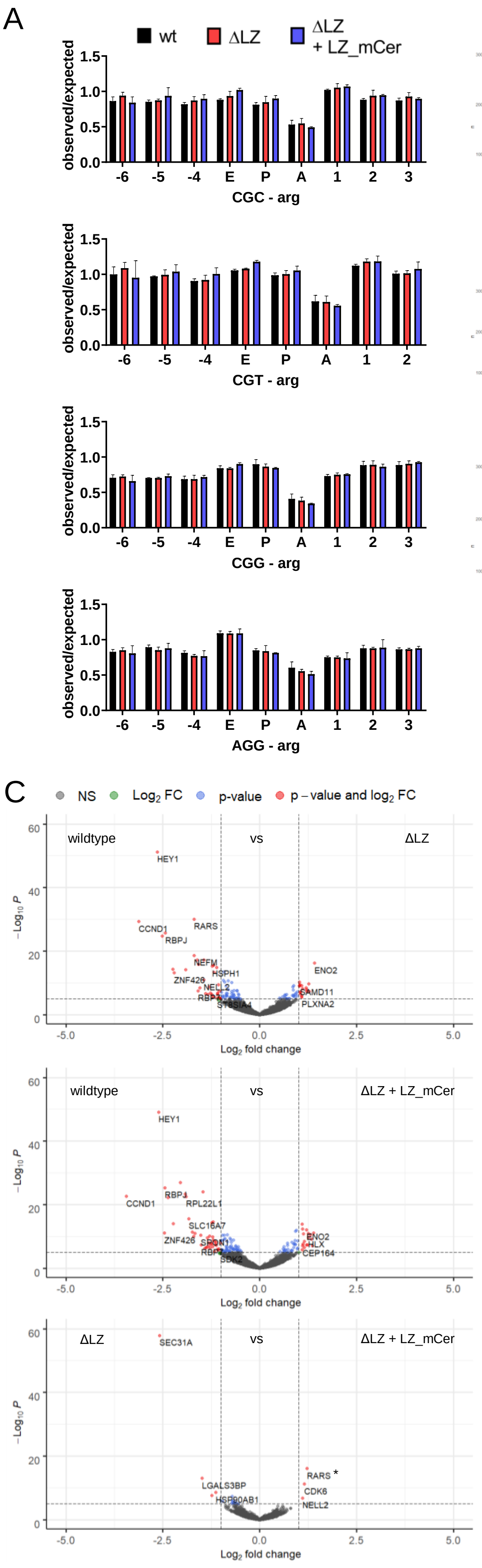

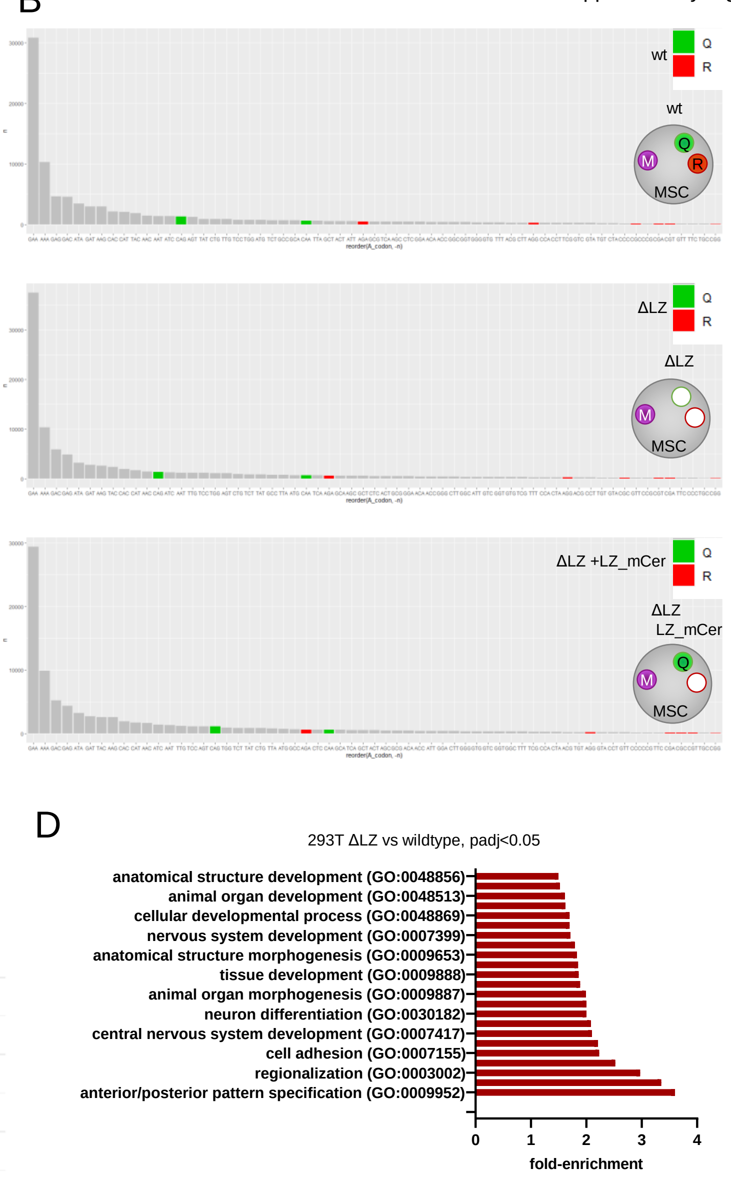

Supplementary Figure 3: Loss of aaRSs from the MSC did not lead to pausing on the cognate codons but some genes are differentially translated. (A) Observed/expected ribosome occupancy on arginine codons as calculated from ribosome profiling did not differ depending on the composition of the MSC (wt: MSC, ΔLZ: MSCΔRQ, ΔLZ + LZ-mCer: MSCΔR). Lack of charged tRNA would lead to a stall at the respective codon and a higher occupancy than expected. (B) Histogram of pauses (P≥10) per codon as calculated from ribosome profiling. Glutamine and arginine codons are highlighted in green (glutamine, Q) and red (arginine, R), respectively. (C) Differential translation (ribosomal occupancy) in ΔLZ or ΔLZ + LZ_mCer cells vs wt. ΔLZ (MSCΔRQ) vs wildtype translation. ΔLZ + LZ_mCer (MSCΔR) vs wt. Vertical line: log2-fold change ±1.0, horizontal line: -log10P 1x106. * Both endogenous RARS1 and the overexpressed LZ_mCer transgene (containing part of RARS1) were counted towards the RARS1 gene. (D) GO analysis of differentially translated genes in ΔLZ versus wt cells. Genes involved in development were enriched. GO terms with FDR P < 0.05 are shown.

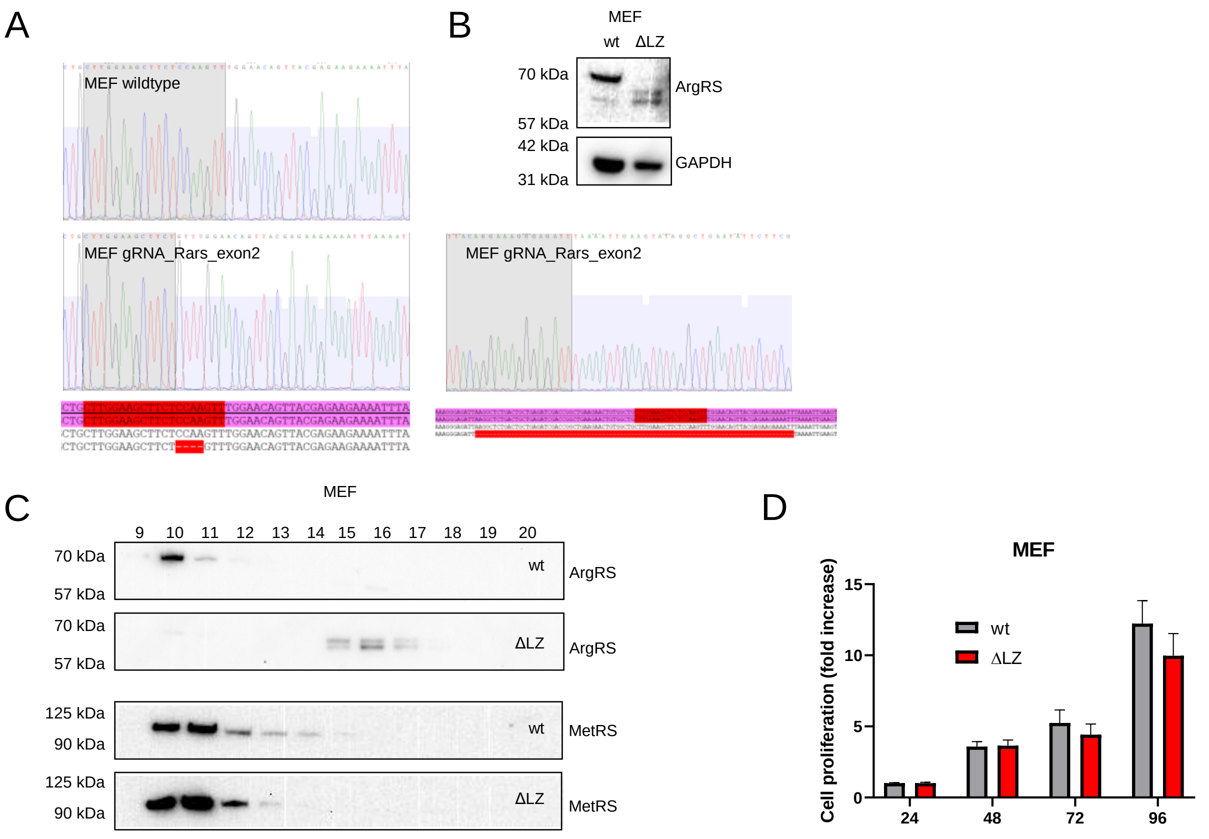

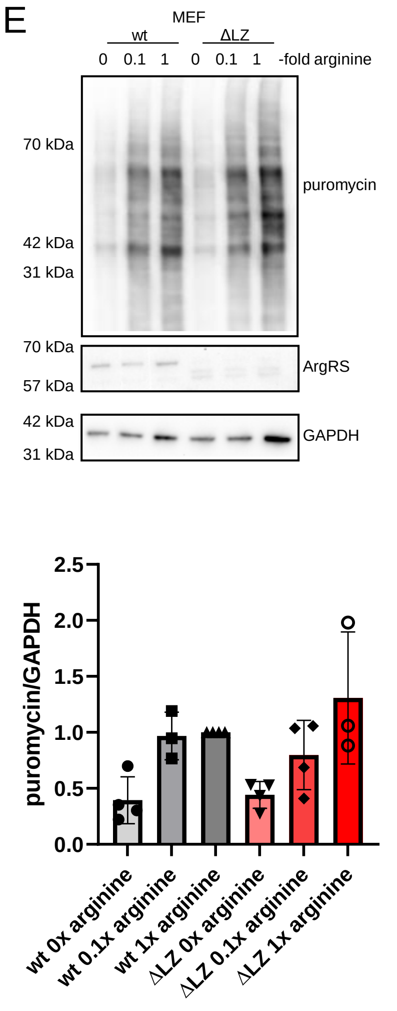

Supplementary Figure 4: Proliferation and protein synthesis in murine embryonic fibroblasts upon exclusion of ArgRS from the MSC. (A) Sanger sequencing of *Rars1* in a MEF cell clone with the desired edit and wildtype. gRNA targeting exon 2 in the murine *Rars1* gene (red) led to the disruption of the gene before the second ATG start codon. Two different modifications were found: The gRNA is shadowed in wildtype and the remaining gRNA sequence in the left clone where the deletion leads to a frame shift. In the same clone, a large 88 bp deletion was also found, here the shadow indicates the side upstream of the deletion. (B) Western blot of ArgRS from cell lysate of wildtype and edited MEFs show loss of full-length murine ArgRS on protein level. The two bands likely originate from the two distinct modifications on each allele. (C) Size exclusion fractionation of MEF cell lysate followed by Western blot detection of aaRS. ArgRS was excluded from the MSC in ΔLZ MEFs. MetRS was retained at higher molecular weight fractions suggesting that the overall integrity of the complex was not affected. (D) Cell viability on different days after seeding was assessed by Alamar Blue. Mean of quadruplets are shown with standard derivation. Cell viability in MEF cells with either wildtype or ΔLZ ArgRS. (E) Puromycin incorporation into MEF cells at different arginine concentrations, detected by Western blot, quantified by densitometry. A representative of 4 repeats is shown. wt 0x vs wt 0.1x: p=0.016, ΔLZ 0x vs ΔLZ 1x: p=0.032, all other n.s.

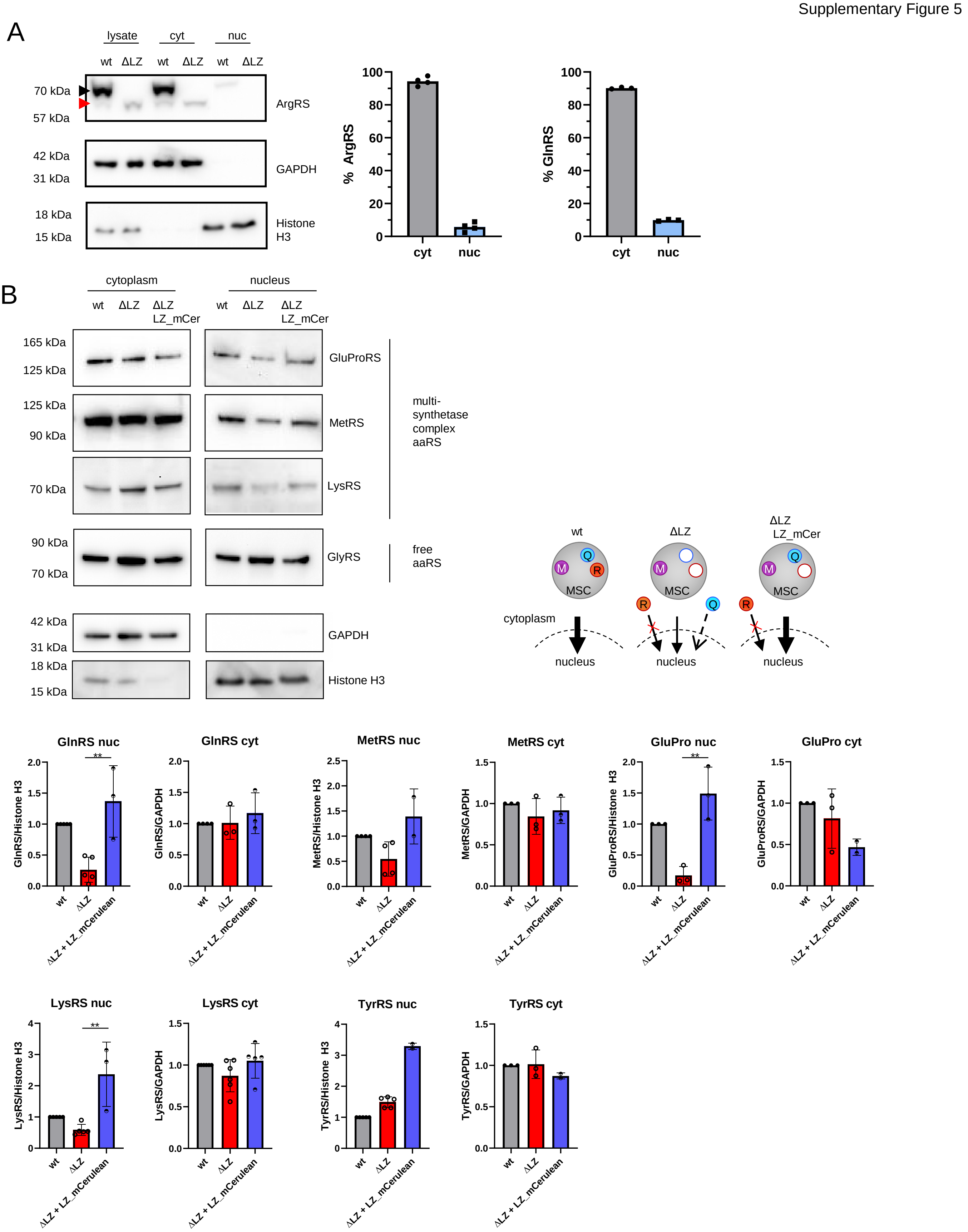

Supplementary Figure 5: Nuclear localization of MSC proteins is increased if the ArgRS LZ and GlnRS are present. (A) Western blot comparing ArgRS levels in whole cell lysate (WCL), cytoplasmic fraction (cyt) and the nuclear fraction (nuc). 2x more sample was loaded of the nuclear fraction after dilution of cytoplasmic and nuclear fraction to equal volumes after cell fractionation. Lysate is the same Western blot as shown in Figure 1A. Densitometric quantification of nuclear and cytoplasmic ArgRS and GlnRS from Western blot, shown as percentage of total ArgRS or GlnRS. (B) Representative Western blots and their densitometric quantification (including for Figure 6A, B) of aaRSs after cell fractionation. Western blots were imaged at different exposures for cytoplasmic and nuclear fractions. Glutaminyl-tRNA synthetase, GlnRS. Methionyl-tRNA synthetase, MetRS. Glutamyl-Prolyl-tRNA synthetase, GluProRS. Lysyl-tRNA synthetase, LysRS. Glycyl-tRNA synthetase, GlyRS. Tyrosyl-tRNA synthetase, TyrRS. (A, B) GAPDH: cytoplasm loading control. Histone H3: nuclear loading control.

Supplementary Tables

Supplementary Table S1: ArgRS peptides retrieved from HEK 293T wildtype or ΔLZ cells.

Supplementary Table S2: Interaction partners of MetRS in HEK 293T wildtype cells.

Supplementary Table S3: Interaction partners of MetRS in HEK 293T ΔLZ cells.

Supplementary Table S4: Number of raw reads, reads after trimming, and reads mapped to human genome after discarding reads from ribosomal rRNAs (60-80 % of all reads).

Supplementary Table S5: Differential ribosome footprint density in HEK293T ΔLZ versus wildtype cells.

Supplementary Table S6: Differential ribosome footprint density in HEK293T ΔLZ + LZ_mCer versus wildtype cells.
